# The association of tumor necrosis factor superfamily 13 with recurrence of immunoglobulin A nephropathy in living related kidney transplantation

**DOI:** 10.1186/s12882-019-1222-4

**Published:** 2019-01-31

**Authors:** Hyung Ah Jo, Seung Seok Han, Sunhwa Lee, Joo Young Kim, Seung Hee Yang, Hajeong Lee, Jae Seok Yang, Jung Pyo Lee, Kwon Wook Joo, Chun Soo Lim, Yon Su Kim, Curie Ahn, Jin Suk Han, Dong Ki Kim

**Affiliations:** 1 Department of Internal Medicine, Seoul National University College of Medicine, Seoul, Republic of Korea; 2 Department of Internal Medicine, Inje University Ilsan Paik Hospital, Ilsan, Republic of Korea; 3 Kidney Research Institute, Seoul National University, Seoul, Republic of Korea; 4 Transplantation Research Institute, Seoul National University College of Medicine, Seoul, Republic of Korea; 5 Department of Surgery, Transplantation Center, Seoul National University Hospital, Seoul, Republic of Korea; 6 Department of Internal Medicine, Seoul National University Boramae Medical Center, Seoul, Republic of Korea

**Keywords:** IgA nephropathy, Tumor necrosis factor superfamily 13, Recurrence

## Abstract

**Background:**

An increasing amount of evidence has demonstrated an association between an increase in the level of tumor necrosis factor superfamily 13 (TNFSF13) and immunoglobulin A nephropathy (IgAN) progression. We aimed to evaluate if the level of pre-transplant serum TNFSF13 is predictive of IgAN recurrence after kidney transplantation.

**Methods:**

This analysis was based on the clinical and laboratory data of 69 patients with IgAN who underwent first kidney transplantation with no evidence of mesangial IgA deposits in zero-time transplantation biopsy. We measured pre-transplant serum TNFSF13, total IgA, and galactose-deficient IgA1 levels.

**Results:**

The recurrence rate of IgAN over a median follow-up duration of 5.1 years was 15.9% (11/69 patients), with a mean time to the first recurrence of 1.7 years. The high pre-transplant TNFSF13 level was associated with IgAN recurrence after kidney transplantation among patients who received a graft from a living related donor.

**Conclusions:**

This study highlights association of TNFSF13 levels in recurrent IgAN patients who undergo living related donor transplantation. Further research is needed to clarify mechanisms by which TNFSF13 affects the recurrence of IgA nephropathy.

## Background

Immunoglobulin A nephropathy (IgAN) is the most common type of primary glomerulonephritis [1, 2], and a significant number of patients progress to end-stage renal disease (ESRD) requiring dialysis or kidney transplantation [3]. In cases of transplant recipients, recurrence of primary glomerulonephritis commonly affects graft and patient outcomes [2]. The recurrence rate of IgAN after kidney transplantation has been estimated to be 9 to 61%, with this wide variability likely to reflect differences in follow-up duration and biopsy policies across institutions [4, 5]. While the impact of the recurrence of IgA deposits on graft function has been debated, recurrent IgAN has been associated with unfavorable outcomes in long-term follow-up studies [2, 6–8]. Unfortunately, however, there is no biomarker that can predict IgAN recurrence in these patients.

Tumor necrosis factor superfamily 13 (TNFSF13), also known as a proliferation inducing ligand (APRIL), is secreted from antigen-presenting cells and is engaged in adaptive and innate immune responses by binding to receptors on B and T cells [9, 10]. An in vivo analysis using *TNFSF13* gene knockout mice showed impaired IgA antibody responses to mucosal immunization [11]. Conversely, *TNFSF13* transgenic mice showed enhanced T-cell independent humoral responses [12]. The *TNFSF13* locus has recently been identified as a susceptibility gene in a Han Chinese genome-wide association study of IgAN patients [13]. Furthermore, high serum TNFSF13 levels in IgAN patients could predict the progression of renal disease based on B cell stimulation [14]. The high recurrence rates after kidney transplantation in IgAN patients suggests an impaired host IgA immune system. Therefore, TNFSF13, which is known to be involved in B cell and immunoglobulin A immune system function, could affect recurrent IgAN. The objective of this study was to find an association between pre-transplant serum TNFSF13 levels and recurrent IgAN.

## Methods

### Patients and data collection

Between January 2011 and October 2015, 80 patients with biopsy-proven IgAN underwent kidney transplantation at our institution. Among these patients, we enrolled 69 who underwent first kidney transplantation and did not show mesangial IgA deposits on biopsy at the time of kidney transplantation and have allograft biopsy data within one year after transplantation. The following information was extracted from medical records for analysis: age, sex, renal replacement therapy, the donor age and sex, number of human leukocyte antigen (HLA) mismatches, ABO mismatch, donor type, desensitization treatment, induction treatments administered, withdrawal of steroid treatment and acute rejection events and trough level of tacrolimus within one year after kidney transplantation. Highly sensitized patients defined as having panel reactive antibody higher than 50% [15]. Allograft failure was defined by the need to re-initiate dialysis or when the estimated glomerular filtration rates (eGFR) fell below 15 mL/min/1.73 m^2^. Clinicopathologic recurrence of IgAN after kidney transplantation was defined as a clinically evident disease with deteriorating renal function and identification of mesangial IgA deposits on immunofluorescence staining with mesangial hypercellularity in allograft biopsy tissue [16]. Renal function decline was defined as eGFR-time slope, differences in eGFR between the peak value in the post-transplant period within 2 months after kidney transplantation and the nadir value at the time of last follow up time divided by follow up duration.

### Laboratory tests

Of the 69 IgAN patients initially enrolled in the study, serum samples at the time of kidney transplantation to quantify pre-transplant TNFSF13 levels were obtained from 62 patients. We also recruited the serum samples from non-ESRD IgAN patients who had eGFR > 30 mL/min/1.73 m^2^. The samples were frozen at − 80 °C until analysis. Serum levels TNFSF13 were quantified using an enzyme-linked immunosorbent assay (ELISA) kit (eBioscience, San Diego, CA, USA), based on previously published methods [14]. Serum Gd-IgA1 levels were quantified using a lectin ELISA with *Helix promatia* agglutinin. Serum Gd-IgA1 levels were expressed in U/mL, where 1 U of Gd-IgA1 was defined as 10.0 μg of standard, and 1 U/mL was expressed as 1 U/ng IgA after normalization to total IgA levels. We purified Gd-IgA1 from an IgAN patient’s plasma using immobilized Jacalin (Thermo Scientific, Rockford, IL, USA) for the Gd-IgA1 method, as previously reported [14].

### Statistical analysis

Comparisons were performed between patients *with* and *without* recurrent IgAN after kidney transplantation. The data are presented as the mean ± standard deviation (S.D) for continuous variables and as proportions for categorical variables. Differences between recurrent and non-recurrent group were evaluated using the t-test for normally distributed continuous variables and Mann-Whitney U test for non-normally distributed continuous variables. The chi-squared or Fisher’s exact test was used for categorical variables. A Cox regression model was used to calculate the unadjusted and adjusted hazard ratios (HRs) and the 95% confidence intervals (CIs) for the factors that were associated with IgAN recurrence after kidney transplantation. The interaction terms between TNFSF13 and adjusted variables were confirmed by Cox analysis. A *p*-value < 0.05 was considered statistically significant. All statistical analyses were performed using IBM®SPSS® software, version 21.0 (IBM Corporation, Armonk, NY, USA).

## Results

### Baseline characteristics and clinical outcomes

**Fig. 1:**
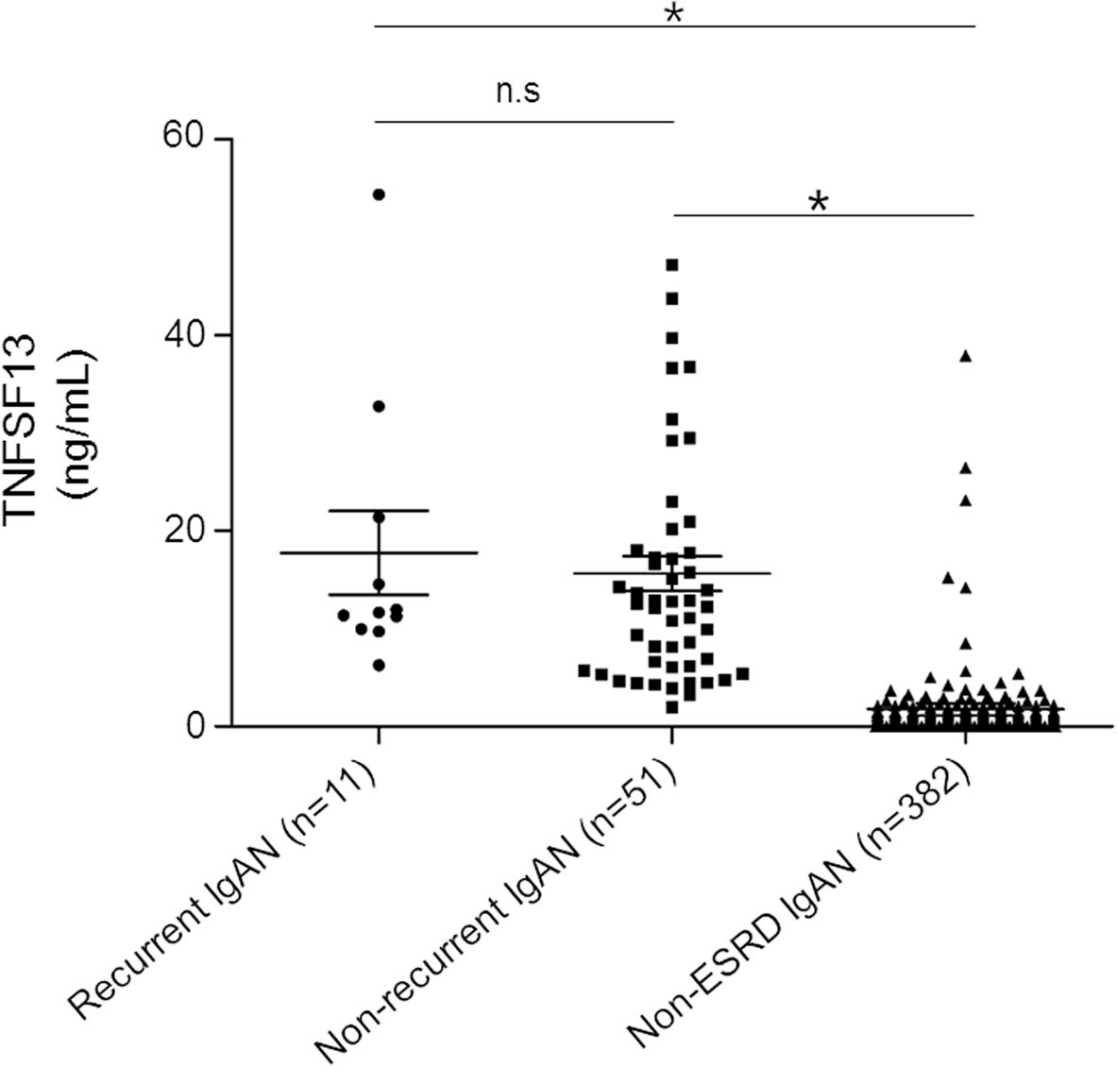
Comparison of serum TNFSF13 levels in patients with recurrent, non-recurrent immunoglobulin A nephropathy and patients with immunoglobulin A nephropathy not on dialysis. *p = 0.000, n.s: non-significant

This study included 40 men (58.0%) and 29 women who underwent first kidney transplantation for ESRD due to biopsy-proven IgAN. At the time of transplantation, their mean age was 43.7 years (Table 1). Over a median follow up of 1865 days (mean, 1835 days), there were no mortalities, and one case of allograft failure caused by the patient discontinuing the immunosuppressive therapy was observed. Recurrent IgAN were identified in 11 patients (15.9%). Among these cases, 3 received deceased donor grafts, 1 received an unrelated living donor graft and the other 7 received related living donor grafts. There was no difference between the recurrent and non-recurrent groups in the proportions of patients treated with tacrolimus, cyclosporine or mycophenolate mofetil. Also, the proportion of patients who received a desensitization treatment and highly sensitized patients who had panel reactive activity over 50% were not different between the groups. The rate of acute rejection was marginally higher among patients *with* than *without* IgAN recurrence (*p* = 0.056). Pre-transplant serum TNFSF13 levels in the study population were significantly higher than in the 382 patients with non-ESRD IgAN (mean 1.75 ± 11.94 ng/mL) (Fig. 1). However, the levels were not different between the recurrent (mean 17.75 ± 14.14 ng/mL) and non-recurrent (mean 15.66 ± 12.79 ng/mL) groups (Fig. 1). Specific HLA types (A2, B35, and DR4) and pre-transplant serum levels of TNFSF13 and galactose deficient IgA1 (Gd-IgA1) were equivalents between groups. The eGFR-time slope was steeper in the recurrent than in the non-recurrent group, although this difference was not statistically significant.

**Table 1:** Baseline characteristics of the study subjects

	All	Recurrence	Non-recurrence	*p*-value
Patients, n	69	11	58	–
Age, years	43.7 ± 13.4	43.4 ± 13.5	43.8 ± 13.5	0.923
Sex (male/female)	40/29	10/1	28/30	0.019
Dialysis/Preemptive kidney transplantation	53/16	9/2	44/14	1.000
Living related donor	36 (52.2)	7 (63.6)	29 (50.0)	0.406
Panel reactive activity > 50%	10 (14.5)	0 (0)	10 (17.2)	0.345
Desensitization treatment	13 (18.8)	1 (9.1)	12 (20.7)	0.676
Number of HLA mismatches	2.88 ± 1.84	2.45 ± 1.97	2.97 ± 1.82	0.354
ABO incompatible transplantation	10 (14.5)	1 (9.1)	9 (15.5)	1.000
Induction treatment (ATG/anti-IL-2)	0/68	0/10	0/58	0.159
Mean follow up duration after transplantation (days)	1835 ± 440	1782 ± 542	1845 ± 423	0.663
Time to transplantation from dialysis (days, IQR)	1387 ± 1611 (84–2109)	1514 ± 1477 (219–2516)	1360 ± 1652 (83–2133)	0.568
Acute rejection	32 (46.4)	8 (72.7)	24 (41.4)	0.056
Mean time of recurrence of IgA on allograft (days, IQR)	–	614 ± 443 (390–726)	–	–
Serum creatinine (at time of recurrence, mg/dL)		2.96 ± 1.52		
eGFR-time slope (delta eGFR/year)	−3.64 ± 4.88	−4.50 ± 3.99	−3.47 ± 5.04	0.302
HLA-A2	30 (43.5)	6 (54.5)	24 (41.4)	0.514
HLA-B35	12 (17.4)	2 (18.2)	10 (17.2)	1.000
HLA-DR4	40 (58.0)	9 (81.8)	31 (53.4)	0.104
Tacrolimus	66 (95.7)	10 (90.9)	56 (96.6)	0.411
Cyclosporine	7 (10.1)	2 (18.2)	5 (8.6)	0.309
Mycophenolate mofetil	53 (76.8)	7 (63.6)	46 (79.3)	0.265
Trough level of tacrolimus within one year from transplantation	8.30 ± 1.70	8.92 ± 1.38	8.19 ± 1.74	0.218
TNFSF13 (ng/mL)	16.03 ± 12.95	17.75 ± 14.14	15.66 ± 12.79	0.525
Gd-IgA1 (units/ng IgA)	5.77 ± 6.79	3.28 ± 1.43	6.35 ± 7.40	0.208

*HLA* human leucocyte antigen, *ATG* anti-thymocyte globulin, *IL-2* interleukin-2, *IQR* interquartile range, *eGFR* estimated glomerular filtration rate, *TNFSF13* Tumor necrosis factor superfamily 13, *Gd-IgA1* Galactose deficient-IgA1

### Correlation between TNFSF13 levels and IgAN recurrence after kidney transplantation among patients who received living related donor grafts

**Fig. 2:**
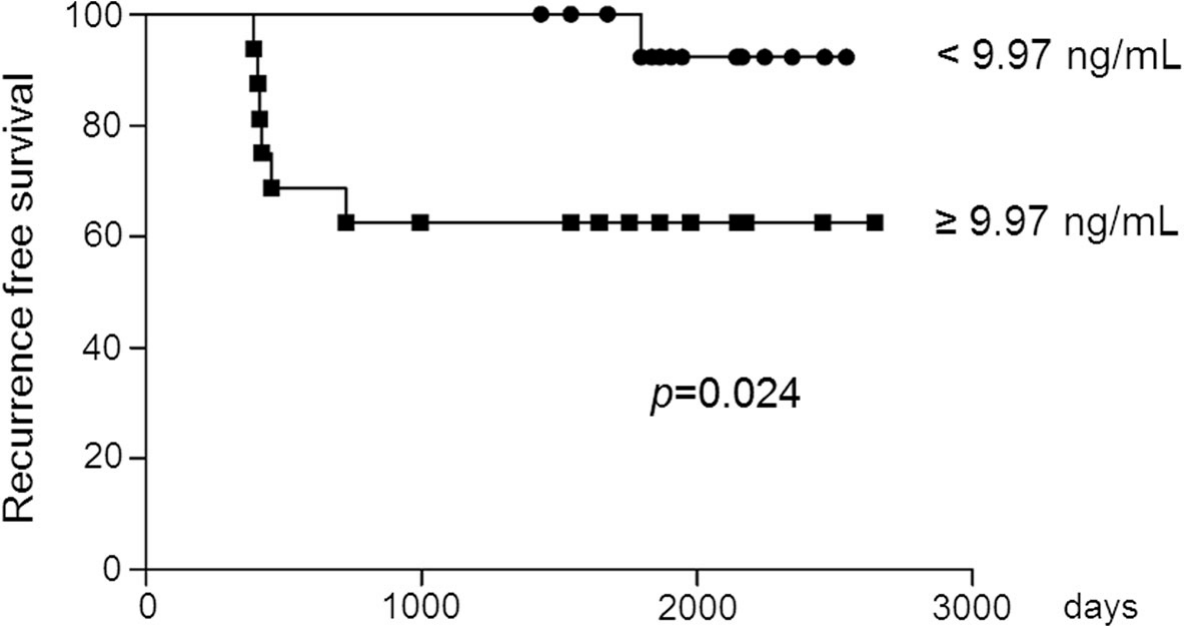
Kaplan-Meier survival curves of recurrence after kidney transplantation in immunoglobulin A nephropathy patients who received living related donor grafts according to the median levels of pre-transplant serum TNFSF13

We did identify a specific association between pre-transplant serum TNFSF13 levels with IgAN recurrence among patients who received living related donor grafts in an interaction analysis (Table 2), though not significant all transplant patients (adjusted HR, 1.111; *p* = 0.620). There was a significant interaction between pre-transplant serum TNFSF13 levels with living related transplantation for recurrent IgAN (*p* = 0.018). Pre-transplant TNFSF13 levels were associated with an increased risk of IgAN recurrence among patients who received a graft from a living related donor after adjusting the recipient’s age and follow up duration (Table 3, HR, 1.685; *p* = 0.025). Patients who received a graft from a living related donor were dichotomized based on the median pre-transplant serum TNFSF13 level (9.97 ng/mL). Recurrence-free survival was significantly lower among patients with a pre-transplant serum TNFSF13 level greater than median values (Fig. 2).

**Table 2:** An interaction analysis between pre-transplant serum TNFSF13 levels and adjusted variables for recurrent IgA nephropathy

Variables	Adjusted HR	95% CI	*p*-value for interaction
Recipient age ≥ 50 years	0.981	0.915–1.052	0.597
Donor age ≥ 50 years	1.017	0.976–1.060	0.427
HLA full matches	1.054	0.960–1.158	0.266
ABO mismatch	0.985	0.830–1.170	0.864
Follow up duration ≥2000 days	1.030	0.993–1.069	0.115
Gd-IgA1 (units/ng IgA)	0.995	0.985–1.006	0.370
Living related donor	1.046	1.008–1.085	0.018

HR: hazard ratio, 95% CI: confidence interval. The hazard ratio was expressed as 1 units of Gd-IgA1/ng IgA increase

**Table 3:** Hazard ratios for recurrent immunoglobulin A nephropathy in patients who underwent kidney transplantation with living-related donor grafts (*n* = 36)

Variables	Unadjusted HR	95% CI	*p*-value	Adjusted HR	95% CI	*p-*value
Recipient age	1.003	0.952–1.057	0.902	1.006	0.947–1.068	0.846
Donor age	0.988	0.924–1.056	0.718			
HLA full matches	2.037	0.394–10.541	0.396			
ABO mismatch	0.554	0.066–4.625	0.586			
Follow up duration	0.999	0.997–1.001	0.332	0.999	0.997–1.001	0.198
Gd-IgA1 (units/ng IgA)	0.881	0.644–1.205	0.427			
TNFSF 13 (10 ng/mL)	1.585	1.030–2.439	0.036	1.685	1.068–2.658	0.025

HR: hazard ratio, 95% CI: confidence interval. The hazard ratio was expressed as 1 units of Gd-IgA1/ng IgA increase, 10 ng/mL TNFSF13 increase

## Discussion

Several factors, including genetic and environmental factors, contribute to the pathogenesis of IgAN. Among the several factors involved in the pathogenesis of IgAN, abnormal mucosal immunity is an important factor for triggering IgAN. As previously noted, TNFSF13 is known to be involved in abnormal mucosal immunity via T-cell independent production of IgA- producing B cells and IgA1 to IgA2 class switching [9, 17–20]. TNFSF13 levels can affect the etiology of recurrent IgAN, based on findings of various studies about the effect of TNFSF13 on the IgA mucosal immune system [11, 14]. It is well known that a longer follow-up period after transplantation and younger age of a recipient at transplantation increase the risk of IgAN recurrence [2]. A previous study reported a high recurrence rate of IgAN after kidney transplantation among recipients with zero-HLA mismatches [21]. In our study, we identified that a higher pre-transplant serum level of TNFSF13 was associated with IgAN recurrence on multivariate analysis among patients who received a graft from a living related donor after adjusting for the recipient’s age at transplantation, the length of follow up duration.

We could not elucidate why there was an association between the levels of TNFSF13 on the recurrent IgAN only in living related donor graft but not in entire subjects. Although the mechanism of increased recurrent IgAN in living related grafts is unclear, several explanations could be possible. First, TNFSF13 shown to be increased the overall IgA secretion, although not specific to Gd-IgA1 [14]. However, unfortunately, we could not confirm that TNFSF13 would affect recurrent IgAN via increasing the relative amount of Gd-IgA1 because there were no differences in the level of Gd-IgA1 between the groups. Second, the pathogenesis of IgAN requires several hits, and we carefully assume there was the role of TNFSF13 in the production of anti-glycan antibody. It is essential for the generation of anti-glycan IgG due to a defect of allorecognition to Gd-IgA1 in the pathogenesis of IgAN [22]. A recent study has shown that TNFSF13 also involved in adaptive immunity through influencing on memory T cells [23]. We could not measure anti-glycan antibodies in the present study; however, we carefully suggest that TNFSF13 would affect recurrent IgAN by engaging in alloantibody response. Third, the similar genetic background in the living related grafts with recipients would have affected the recurrent IgAN [24]. It can be inferred that the mesangial immune complex deposition of living related grafts is more likely to occur [25, 26]. TNFSF13 may have upregulated the production of systemic IgA or pathognomonic IgA1-immune complex, and these factors may be profound in living related donor grafts having the genetic susceptibility. This issue would be resolved by the further experimental investigation.

Although IgAN was traditionally considered as a benign disease, more recent evidence has demonstrated that recurrence of IgAN in renal allografts is associated with unfavorable outcomes in graft function [2, 8, 27, 28]. In this study, we observed only one event of graft failure due to the self-discontinuing steroid. Therefore, we did not find an association between serum TNFSF13 levels and graft outcome. Additionally, we did not identify an HLA-specific effect, as reported in previous studies, including a negative effect for HLA-B35 and DR4 and a protective effect for HLA-A2 [6, 29]. As the majority of the patients in our study group received anti-interleukin 2 for induction therapy, we were unable to demonstrate the protective role of antithymocyte globulin induction therapy on IgAN recurrence, as reported in a previous study [30].

According to Australia and New Zealand Dialysis and Transplant (ANZDATA) registry, which has accumulated data over 30 years, showed that recurrence of IgAN was 5.1, 15% at 5, 15 years after kidney transplantation [2]. The rate of IgAN recurrence in our study was 15.9% over a median follow up duration of 5.1 years, a rate which was higher than the rate based on the ANZDATA registry data but lower than recurrence rate of 29–61% reported in other studies [4, 29]. Differences in reported rates of IgAN recurrence might result from differences in biopsy policy between institutions and differences in the length of follow up. Further prospective studies with sufficient follow-up duration are needed to clarify the association between pre-transplant serum TNFSF13 levels and the recurrence rate and graft functionality in IgAN patients.

The present study has several limitations. The number of total subjects and events in the present study was too small to obtain the predictive value of TNFSF13 for recurrent IgAN. Relatively shorter follow up duration was also a limitation of the present study. TNFSF13 was only associated with living related transplantation, and the predictive value was not shown. Further studies in the sufficient number of cohorts are needed to confirm these findings and to show cut off values for the predictive value of TNFSF13.

In summary, we identified an association between pre-transplant serum TNFSF13 levels and IgAN recurrence among patients who received a graft from a living related donor. Our findings suggest that there may be a possible role of TNFSF13 in IgAN recurrence among patients who undergo living related donor graft transplantation.

## Conclusions

This study provides evidence to suggest that pre-transplant serum TNFSF13 levels is associated with recurrent IgAN who had living related transplantation. Regarding the concern of recurrence of IgAN on graft function, these results require further studies to validate the role of TNFSF13 in the recurrence of IgAN.

## Abbreviations

anti-IL-2: anti-interleukin 2
APRIL: A proliferation-inducing ligand
ATG: Antithymocyte globulin
eGFR: Estimated glomerular filtration rates
ESRD: End-stage renal disease
Gd-IgA1: Galactose deficient IgA1
HLA: Human leucocyte antigen
HR: Hazard ratio
IgAN: Immunoglobulin A nephropathy
TNFSF13: Tumor necrosis factor superfamily
